# Characterization of Semolina and Pasta Obtained from Hard Hexaploid Wheat (*Triticum aestivum* L.) Developed Through Selection Assisted by Molecular Markers

**DOI:** 10.3390/foods14111990

**Published:** 2025-06-05

**Authors:** María B. Vignola, Mariela C. Bustos, Leonardo Vanzetti, Alfonsina E. Andreatta, Gabriela T. Pérez

**Affiliations:** 1 Ingeniería de Procesos Sustentables (InProSus), Facultad Regional San Francisco, Universidad Tecnológica Nacional, Av. de la Universidad 501, San Francisco CP 2400, Córdoba, Argentina; mvignola@facultad.sanfrancisco.utn.edu.ar (M.B.V.); aandreatta@facultad.sanfrancisco.utn.edu.ar (A.E.A.); 2 Consejo Nacional de Investigaciones Científicas y Técnicas (CONICET), Godoy Cruz CP 2290, Buenos Aires, Argentina; leonardo.vanzetti@gmail.com; 3 Instituto de Ciencia y Tecnología de los Alimentos Córdoba (ICYTAC, CONICET-UNC), Av. Filloy s/n, Córdoba CP 5000, Argentina; mbustos@agro.unc.edu.ar; 4 Instituto Nacional de Investigación Agropecuaria (INIA), Est. Exp. La Estanzuela, Ruta 50 km 11.5, Colonia CP 70000, Uruguay

**Keywords:** hard hexaploid lines, pasta quality, cooking properties, textural attributes

## Abstract

This study evaluates the potential of hard hexaploid wheat (*Triticum aestivum* L.) lines, developed through marker-assisted selection (MAS), as an alternative to durum wheat for pasta production. Using hard hexaploid lines (SD lines) with targeted traits, such as increased gluten strength, protein content, and yellow coloration, the objective was to assess their performance relative to traditional durum wheat. Results indicate that some hard hexaploid lines demonstrate competitive properties compared to durum wheat genotypes, including protein content exceeding 11.5%, gluten index above 90%, and line SD 55 presented acceptable cooking performance with minimal cooking loss. Although some textural properties like hardness and chewiness were slightly lower than durum pasta, the line SD 34 exhibited characteristics most similar to durum wheat pasta. This study supports MAS-developed bread wheat as a feasible and cost-effective alternative for high-quality pasta production, particularly in regions where durum wheat is less accessible.

## 1. Introduction

Pasta is a popular staple food known for its sensory and nutritional value, convenience, and versatility. Approximately 16.9 million tons of pasta are produced annually worldwide [1]. Pasta is usually made from semolina obtained from the milling of durum wheat (*Triticum turgidum* L. var. durum Desf.) and the addition of water with mixing, followed by extrusion/lamination and either drying or consumption as a fresh product [2]. Among the constituents suitable for pasta production, durum wheat semolina stands out due to its pronounced vitreousness, high protein and carotenoid levels, lower lipoxygenase activity, and gluten composition characterized by limited extensibility and elasticity [3,4].

Starch and proteins are two crucial macromolecular components of flour, and their content and characteristics influence the rheological properties of dough; consequently, the quality of the finished products [5]. Starch, which constitutes about 80% of durum semolina, comprises two main types of glucose polymers: amylose (25–28%) and amylopectin (72–75%). Amylose, an essentially linear polymer, significantly impacts the characteristics of cooked starch and flour pastes [6]. In wheat starches, amylopectin enhances water absorption, swelling, and stickiness of starch granules, while amylose and lipids tend to delay these processes [7]. The pasting properties of starch are important indicators of its suitability for various end uses. Gluten quality is crucial in pasta manufacturing and cooking quality, making it a vital selection criterion in cultivar development [6].

In addition to starch and gluten, non-starch polysaccharides, such as arabinoxylans, also contribute to the functional and nutritional quality of wheat flours. Wheat grains contain these polysaccharides (also referred to as pentosans), which represent approximately 20–27% of the aleurone layer, 23–32% of the bran, and 2–4% of the endosperm. Based on their solubility, arabinoxylans are classified as water-unextractable (WUAX) or water-extractable (WEAX), each with distinct effects on dough properties, particularly in bread making. Their higher water affinity among other dough constituents ultimately increases the water absorbing capacity of flour, which alters dough behavior during handling and processing. Furthermore, as part of dietary fiber, they also have a significant impact on the nutritional quality of cereal-based foods [8].

Durum wheat accounts for only about 5% of global wheat production due to specific climate requirements, making it unsuitable for extremely hot or cold conditions. The widespread success of bread wheat (*Triticum aestivum* L.) is attributed to the plasticity of its hexaploid genome compared to tetraploid wheat [9]. In Argentina, over 98% of wheat production is bread wheat, primarily hard red spring, with 120 cultivars in the market; the remaining 2% includes durum, soft, and waxy wheats [10].

In Argentina, durum wheat cultivation is challenging due to its specific climatic requirements and is mainly limited to certain regions, particularly in the southeast of Buenos Aires. The long distances between these production areas and other key regions, such as Córdoba, result in high transportation costs. This situation is similar across the country, as the significant distances from Buenos Aires make durum wheat more expensive and logistically challenging to obtain for pasta production. As a result, it has become common to find pasta made from bread wheat flour, especially in those products intended for consumers who prioritize cost over quality or in regions distant from the durum wheat cultivation zones [11].

Conventional breeding has played a critical role in wheat improvement in the last few decades. The process of selecting cultivars based on specific genes of interest through the use of molecular markers is referred to as marker-assisted selection (MAS). MAS contributes to overcoming the shortcomings of traditional breeding based on phenotype and greatly improves crop breeding efficiency for pyramiding multi-allelic traits. Moreover, it has many advantages over conventional breeding techniques [4,12]. MAS is a technique for achieving the incorporation of desirable gene alleles into bread wheat germplasm. These genes can confer characteristics such as grain hardness, gluten strength, protein content, and flour pigmentation, thus enhancing the quality and functional properties of pasta made from genetically modified bread wheat. The aim of the following study was to evaluate the properties of semolina produced by hard bread wheat developed through MAS for their subsequent use in pasta production.

## 2. Materials and Methods

### 2.1. Developed Germplasm

For the development of bread wheat (*Triticum aestivum* L.) germplasm suitable for pasta production (hard hexaploid lines), the following genotypes were used in an INTA (National Institute of Agricultural Technology) breeding program with the aim of pyramiding genes of interest:

1. Chinese Spring Red Egyptian line is a hexaploid wheat line that carries a deletion on chromosome 5D, where the main genes related to grain texture, known as puroindolins, *PinA-D1*, and *PinB-D1*, are located [13].
2. ProINTA Gaucho is an Argentine hexaploid wheat variety that exhibits a translocation on chromosome 7A, carrying a segment of chromosome 7E from *Lophopyrum ponticum*. In this translocation are located resistance genes to rust *Lr19* and the *Psy-E1* gene, which imparts yellow coloration to the flour [14].
3. BC6F3 line of the Argentinean variety BIOINTA 3000 incorporates a segment containing the *GPC-B1* gene from *Triticum turgidum* ssp. dicoccoides on chromosome 6B. This gene is involved in senescence and nutrient remobilization to the grain, resulting in an increase in protein content [15]. Additionally, the BIOINTA 3000 variety carries the High Molecular Weight Glutenin allele *Glu-B1al*, also known as the *Glu-B1 7* overexpressed allele (*Glu-B1 7oe*). This allele has been strongly associated with improving gluten strength in genotypes possessing it [16].

#### 2.1.1. Breeding Scheme and Marker-Assisted Selection

**Figure 1:**
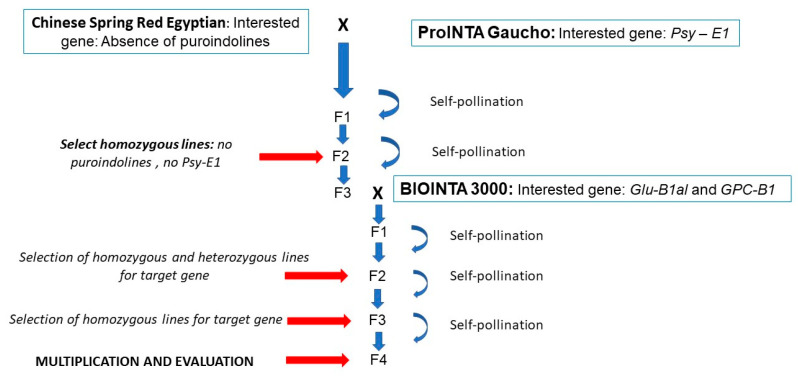
Diagram of crossbreeding and selection used for the development of hard hexaploid lines suitable for pasta making.

Lines with the desired gene stack for pasta production were developed following the breeding scheme outlined in Figure 1.

#### 2.1.2. Molecular Markers Used in the MAS Process

Genomic DNA was extracted from fresh leaves of individual plants at a three-leaf stage using a fast, small-scale DNA isolation procedure based on Helguera et al. [17]. To detect the deletion of the puroindolines genes, associated with high grain hardness, the marker for the *PinB-D1* gene developed by Gautier et al. [18] was used. The absence of amplification indicates the presence of puroindolines deletion in a homozygous state. For the detection of the *Glu-B1al* (*7oe*) allele, associated with high tenacity gluten, the co-dominant functional marker developed by Butow et al. [19] was used. For the *GPC-B1* gene transferred from *Triticum turgidum* ssp. *Dicoccoides* codominant molecular marker Xuhw89, developed by Distelfeld et al. [20], was used to follow the High Grain Protein Content gene in the breeding process. Finally, for the 7EL segment containing *Psy-E1* and *Lr19* genes, dominant marker developed by Prins et al. [21] was used to follow the flour yellow pigment in the breeding process.

#### 2.1.3. Field Trials and Agronomic Performance

Lines with the target genes in a homozygous state were multiplied for genotype evaluation based on pasta quality parameters. Four developed genotypes, along with the three parental lines used in the crosses, were planted in field plots measuring 1.2 × 5 m, employing a Randomized Complete Block Design (RCBD) with 2 replications in Córdoba, Argentina. The trial also included four cultivars of durum wheat with varying qualities as reference standards for industrial parameters. The trial was sown at a density of 350 plants per square meter using an experimental planter and harvested at maturity. Eleven samples from the 2017 harvest were evaluated (Table 1).

### 2.2. Grain Vitreousness and Milling

The percentage of vitreousness of the grains was determined using the diaphanoscope method [22]. The sample size of the grains to be milled was approximately 500 g. The grains were conditioned to 14% moisture prior to milling. The milling was carried out in a Buhler 202 D mill following the IRAM 15854 standards for grain conditioning and milling [23]. The semolina yield ranged between 60% and 74%.

### 2.3. Particle Size Distribution

Semolina particle size distribution was determined with a laser particle size analyzer (LA-960, Horiba Instruments, Irvine, CA, USA). The diameter of the semolina particles was measured based on the volume distribution, taking the mean particle volume (VMD) calculated using the Fraunhofer theory. The average, D(10), D(50) (value of the particle diameter at 50% in the cumulative distribution), D(90), d[4,3] (volume-weighted mean diameter of particles) and span (Span = [D(90)− D(10)]/(D50)), which reflects the distribution of particles around the median particle size values, were reported in µm (HORIBA LA960 for windows [Wet] 2.20). The latter (span) provides the separation range between 10 and 90% of the points, normalized to the midpoint [24].

### 2.4. Chemical Analysis

The following determinations were made moisture (method 44-19, [25]), ash (method 08-01, [25]), total arabinoxylans (TAx) and water-extractable arabinoxylans (WEAx) [26]; protein content (Method 46-13, [25]), wet gluten (WG) (method 38-10, [25]), dry gluten (DG) and gluten index (GI) (method 38-12, [25]). The last two were analyzed using a Glutomatic 2200 (Perten Instruments, Warriewood, NSW, Australia). Total starch content and amylose content of starch were determined by the method of Gibson et al. [27] using a Total Starch Assay Kit (AA/AMG) and an amylopectin–amylose assay kit (Megazyme Int. Ireland Ltd., Wicklow, Ireland).

### 2.5. Starch Pasting Properties

Pasting properties of semolina samples were measured using an RVA (Tecmaster, Perten Instruments, Warriewood, NSW, Australia), according to the method 76-1 [25]. Pasting temperature (PT), peak viscosity (PV), final viscosity (FV), trough, breakdown viscosity (BV), and setback were obtained from the pasting curves.

### 2.6. Solvent Retention Capacity

The solvent retention capacity (SRC) of flours was evaluated according to Method 56-11 [25]. Thus, 5 g of the sample was weighed in a 50 mL centrifuge tube. Then, 25 mL of each solvent—water (SRCW), 50% sucrose (SRCSuc), 5% sodium carbonate (SRSCarb), and 5% lactic acid (SRCLac)—was added separately to the samples, and the mixtures were vigorously shaken for 5 s. The mixtures were then shaken every 5 min for 20 min and centrifuged for 15 min at 1000× *g* (at room temperature, 25 °C). The SRC was calculated as the weight of solvent held by samples after centrifugation, supernatant separation, and gel drainage for 10 min, and expressed as a percentage of sample weight, based on each sample’s humidity.

### 2.7. Pasta Making

All pasta samples were prepared according to Vignola et al. [28] with modifications. Each pasta sample was prepared using 50 g of semolina. The moisture content of the pasta dough was approximately 30%. The dough was shaped through the die to obtain a fettuccini form (width of 5 ± 0.2 mm) in a home pasta extruder PE-MP001R (Peabody, Buenos Aires, Argentina). Pasta was then dried at 45 °C in a humidity-controlled (75%) drier for 17.5 h, resulting in a final moisture content of 11.5 ± 0.5 g/100 g. The samples were wrapped in clean film and stored in airtight containers at room temperature until needed.

### 2.8. Pasta Cooking Properties

The following pasta properties were evaluated: optimal cooking time (OCT) (method 16-50, [25]); water absorption (WA), swelling index (SI), cooking loss (CL) according to Tudoricǎ et al. [29]. All determinations were conducted in duplicate.

### 2.9. Cooked Pasta Textural Analysis

The texture of cooked pasta was measured using a texture analyzer (Universal Testing Machine, INSTRON 3342, Norwood, MA, USA) with a 500 N load cell and Texture Expert Software (v1.22, Stable Micro Systems, UK). Parameters determined included pasta hardness, cohesiveness, chewiness, and elasticity. All samples were cooked to their optimum cooking time on the testing day, and excess water was blotted with absorbent paper before testing. A cylindrical probe compressed one 5 cm strand at a rate of 2.5 mm/s to 60% strain, followed by a second compression after 10 s. Five replicates were performed in each pasta sample.

### 2.10. Color of Semolina and Pasta

The color of semolina and cooked pasta was determined with a Minolta 508 d spectrophotometer (Ramsey, NJ, USA). Eight-millimeter measurement apertures, D65 illuminant, 10° angle of observer were set, according to approved methods 14-22 [25]. At least eight readings were taken from semolina and pasta samples. Cooked pasta strands (at least 8) were placed in placed side-by-side on a paper towel to take the readings. They were recorded as CIE-LAB, L* (lightness), a* (redness-greenness), and b* (yellowness–blueness) values [30].

### 2.11. Statistical Analysis

Statistical analyses were performed using Infostat/Professional statistical software (Version 2020e) [31]. Data were examined by ANOVA, and results were compared by Fisher’s test at a significance level of 0.05. The differences among semolina and pasta samples were analyzed, and the results were expressed as the mean of two repetitions. A Principal Component Analysis (PCA) biplot was generated using selected parameters, including particle size distribution, pasta color, textural properties, cooking characteristics, protein content of both semolina and pasta, and WG, DG, to assess the variability among different wheat varieties.

## 3. Results and Discussion

The physicochemical properties (grain vitreousness, particle size distribution, and chemical components) of hard hexaploid lines (SD lines) were compared among them and with the mean values of control durum wheat varieties.

### 3.1. Grain Vitreousness

Vitreousness is a crucial quality factor used internationally for grading durum wheat in different markets. Vitreousness values of semolina samples (hard hexaploid lines, parent lines, and control durum wheats) presented a wider variation from 0 to 85.13% (Table 2). The control durum wheats were the ones that showed the highest values of vitreousness, indicating statistically significant differences (*p* < 0.05). Parent cultivars showed very low vitreousness values, while hard hexaploid lines gave vitreousness values of 0. Several results were reported in the literature. For instance, Kaliniewicz et al. [32], who worked with 12 wheat cultivars divided into four kernel hardness groups (medium soft, medium hard, hard, and very hard), reported vitreous kernel percentages in wheat ranging from 0.6% to 89.4% while Ei-Khayat et al. [33] found vitreousness values between 45% and 93% in six Syrian spring durum wheat cultivars. Vitreous kernels are also considered to have a positive effect on the color and cooking quality of pasta. However, that does not necessarily mean that semolina derived from vitreous grains always produces pasta of good cooking quality [34].

### 3.2. Particle Size Distribution

Table 2 shows the results of the particle size distribution of hard hexaploid lines and the mean value of control durum wheats. SD lines presented higher D(10) values in comparison to control durum wheat, although statistically significant differences were observed only for SD 31 (*p* < 0.05). The particle size distribution of the semolina D(50) fraction was between 283.60 and 310.46 µm, and the mean particle size (d[4,3]) ranged from 295.14 to 547.70 µm. SD 31 showed the highest D(10), D(50), D(90), and d[4,3] values (*p* < 0.05) while the other hexaploid lines did not show a significant difference from the durum wheat mean value. D(90) of SD 31 was more than twice that of the other samples, indicating that larger particle sizes were produced during milling. This suggests that the internal structure of the grain influenced fragmentation, as previously reported by Arriaga et al. [35]. Semolina particle size is a key factor in pasta making. Particle size affects the rate of hydration of the milled product during pasta processing. Several authors indicate how particle size affects not only the hardness of the pasta but also its technological properties, such as optimal cooking time, cooking loss, water absorption, and pasta color [2,36]. For decades, pasta manufacturers used durum wheat flour with a particle size of between 125 to 630 µm [37]. However, in more recent years, pasta manufacturers are using semolina with a finer particle size (125 to 425 µm). Fine semolina is preferred by the pasta industry as it provides higher milling yields, ensures a high and uniform hydration rate, and facilitates the mixing process [38].

### 3.3. Chemical Analysis

According to the International Food Code, the maximum ash content allowed in durum wheat semolina should be 1.3% on a dry basis [39]. The ash content of all genotypes (control durum wheat genotypes and hard hexaploid lines) under study falls within the established limits (1.03–1.21%) (Table 3). Several authors have found lower ash values than those presented in this study [40,41]. The modified hexaploid wheat lines resemble the durum wheat control lines in ash content, with no significant differences observed (Table 3).

TAx content of the semolina samples ranged from 6.58% to 8.24%, while the content of WEAx ranged from 0.40% to 0.50%. The WEAX results obtained in the analyzed semolina samples agree with Arif et al. [8]. However, Maeda et al. [42] observed higher values of WEAx (0.73% and 1.28%) compared to those found in our semolina study. Nevertheless, several researchers have reported lower contents of TAx and WEAx [43,44]. No statistical differences were recorded between the mean value of hard hexaploid lines and the control cultivars of durum wheat for TAx and WEAx contents. Since arabinoxylans are the major polymers in the cell wall, therefore, the main component of dietary fiber in the grain, this component can be used as an estimator of the total dietary fiber content [45].

The starch content in the semolina is crucial for determining the quality of the resulting pasta [46]. The total starch content in the studied semolina samples ranged from 69.90% to 72.95%, showing higher values than those reported in the literature [33,47]. The total starch values observed are consistent with those characteristic of high-quality semolina for pasta production (68.1–71.4%), as indicated by Sissons et al. [48] and De Cindio and Baldino [49]. No statistically significant differences were found in total starch content between the various analyzed hard hexaploid lines and control durum wheats (Table 3).

The amylose content varied between 20.84% and 27.55% (Table 3) and is in concordance with previous studies [50,51]. SD 31 and SD 55 lines recorded the highest amylose values, showing no significant differences compared to the control durum wheats (*p* < 0.05) (Table 3). The ratio of amylose to amylopectin content plays a crucial role in determining pasting and thermal properties, gelatinization, and cooking quality of wheat-based products [7,51]. An amylose/amylopectin ratio corresponding to approximately 18.5–21.5% amylose (i.e., a ratio of about 0.23–0.28) has been shown to produce pasta with optimal firmness, minimal cooking loss, and superior sensory quality [52].

The protein content of semolina influences the technological and nutritional value of semolina and pasta. According to Codex Alimentarius International Food Standards, the minimum protein content of durum wheat semolina should be 11.5% [39,40]. In our study, the protein content of the wheat samples ranged from 9.80% to 11.80% (Table 3). These findings are consistent with similar studies conducted by other researchers [33,41,53]. However, several authors have reported higher protein content in durum wheat cultivars [33,40]. These results collectively demonstrate the variability in protein content across different studies and support the notion that protein content in wheat can vary depending on the specific cultivar, accession, and environment under investigation. Significant differences were found in the protein content among the analyzed cultivars (*p* < 0.05) (Table 3). All modified hexaploid wheat lines showed significantly higher protein contents compared to the control durum wheats, which could make this type of wheat suitable for pasta production. Among the varieties studied, SD 31 had the highest protein content (11.80%), while control durum wheats recorded the lowest contents (9.80%). The parent line BIOINTA 3000 GPC exhibited a mean protein content of 10.83%, significantly differing from the control lines and similar to hard hexaploid lines, indicating that *GPC-B1* gene transfer occurred successfully (Table 3).

Gluten, an important indicator of wheat pasta quality, is an elastic protein showing the suitability of flour for pasta making. Among the wheat quality components, gluten plays the most important role in determining industrial use, and therefore, gluten strength is one of the parameters for classification of wheat for use in bread, cakes, and pasta [40]. The WG content of the analyzed semolina samples exhibited a range of medium values of 25.96% to 31.76% (Table 3) and is in concordance with different authors [40,54,55]. The DG values in the analyzed semolina varied between 9.53% and 12.09%, in agreement with various authors [51,55]. No significant differences were found between hard hexaploid lines and control durum wheats for both WG and DG. Based on the established criteria for high—quality durum—wheat semolina (wet gluten 28–32% and dry gluten > 11%), the WG and DG contents of lines SD 31, SD 34 y SD 55 fall squarely within these ranges and are therefore adequate to produce pasta of good technological quality [56].

The GI is a measurement of wheat proteins that provides a simultaneous determination of gluten quality and quantity. Indeed, GI is a criterion defining whether the gluten quality is weak (GI < 30%), normal (GI = 30–80%), or strong (GI > 80%) [57]. The GI values of the semolina analyzed ranged from 88.02% to 94.47% (Table 3). Fanari et al. [58] reported that the GI values on durum wheat semolina varied between 47.12% and 88%. Kaplan Evlice [55] evaluated 24 genotypes and found GI values ranging from 0.8% to 99.5%, with a mean of 67.5%. These findings highlight the variations in GI values among different wheat varieties and emphasize the importance of GI as an indicator of gluten strength and quality [59]. No statistically significant differences were found among each hard hexaploid lines and control durum wheats (Table 3). However, we observed high GI values in the hard hexaploid lines in comparison to control durum wheats (93.40% vs. 88.02%, *p* < 0.05), exceeding 90%. This is a relevant trait for pasta production, highlighting the potential suitability of these cultivars [60]. The parent line BIOINTA 3000 GPC recorded higher GI values than control durum wheats and similar to hard hexaploid lines, indicating the successful transfer of the specific gene. A higher GI value indicates stronger gluten (≥80%), which is desirable in various food applications that require good gluten development. This is because strong gluten retains the starch molecules during cooking, with the result that the surface of the pasta does not become sticky and the pasta keeps its shape [60].

### 3.4. Color of Semolina

**Figure 2:**
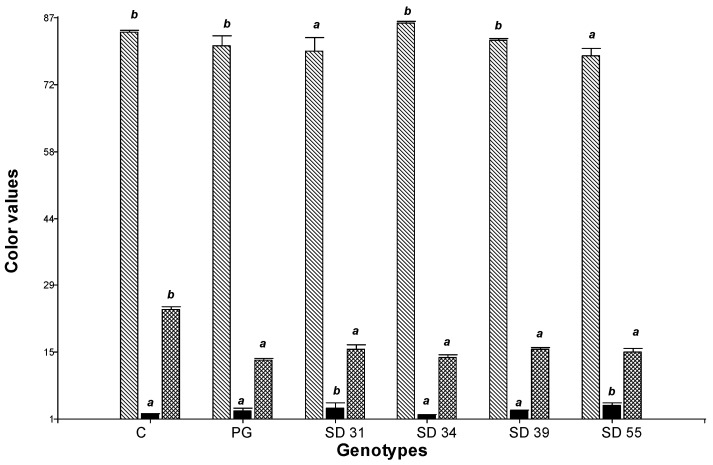
Color (L*, a*, and b* values) of semolina samples. L*: 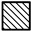, a*: 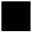 and b*: 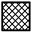. C: control durum wheat, PG: Prointa Gaucho. Different letters on bars corresponding to the same color parameter (L*, a*, or b*) indicate significant differences among genotypes (*p* < 0.05).

Pasta’s appearance can vary from bright yellow to dull brown. Brightness and yellowness of pasta are important quality parameters for pasta companies and consumers [40]. Color values (L*: lightness/brightness, a*: redness b*: yellowness) of semolina samples from different varieties (control durum wheats, hard hexaploid lines and parent line Prointa Gaucho) ranged from 72.89 to 85.74, 0.84 to 4.96, and 13.13 to 26.89, respectively (Figure 2). The findings were consistent with Schulthess et al. [61] and Yildirim and Atasoy [40] who found similar b* and L* values, respectively, in durum wheat varieties. Statistically significant differences were found among the three-color parameters analyzed (*p* < 0.05). SD 34 and SD 39 lines recorded higher L* values and lower a* values, differing statistically significantly from the other SD lines (*p* < 0.05), resembling those recorded for the control durum wheats (L*: 83.43; a*: 1.69). Control durum wheats had higher L* and b* values (more yellowness), which might be due to the presence of a higher amount of carotenoids and xanthophylls [62]. The cultivar Prointa Gaucho, the donor parent of the yellow pigment, exhibited similar values for all parameters to the hard hexaploid lines, indicating successful transfer of the yellow color to the hard hexaploid wheat lines. However, the b* parameter values did not reach those of commercial durum wheats. High-quality pasta is generally characterized by b* values ≥ 20, reflecting its typical yellow coloration [48].

### 3.5. Pasting Properties of Semolina Samples

The cooking quality of pasta is highly influenced by starch gelatinization and protein network formation [63]. While much research has focused on the role of proteins, starch, which constitutes approximately 70–80% of durum wheat semolina, has received comparatively less attention [64]. The Rapid Visco Analyzer (RVA) is widely used to evaluate starch properties by measuring viscosity changes during heating, holding, and cooling cycles, offering insights into molecular transformations that impact pasta quality [64]. Viscograms obtained through RVA provide valuable data on starch gelatinization and retrogradation, which are crucial for predicting cooking behavior and the final texture of pasta [65].

Pasting properties (peak viscosity, trough, breakdown, final viscosity, setback, and pasting temperature) of semolina from different control durum wheats and hard hexaploid lines were shown in Table 4. Similar values for all parameters were found in different semolina samples by Laurent et al. [66] and Wood [67]. Significant differences were observed among the analyzed samples for all evaluated parameters (*p* < 0.05). The SD 31 line recorded the highest values for PV and BV. For FV and setback, the SD line 31 showed the highest value, together with SD 55 and control durum wheats. These same lines also recorded amylose values similar to those of control durum wheats. Higher amylose content generally leads to a greater setback, as amylose has a higher tendency to retrograde, forming more ordered and crystalline structures upon cooling. Additionally, the FV tends to be higher in starches with greater amylose content, as the linear structure of amylose facilitates the formation of a firmer three-dimensional network upon cooling, contributing to higher viscosity [68,69].

Stability of hot starch pastes is described by BV, which increases when starch granules become weakened in order to facilitate disruption of granular structure [70]. Significant differences were found among the analyzed semolina (*p* < 0.05) (Table 4). All modified hexaploid wheat lines exhibited higher values of BV. SD 31 was the line that showed the significantly highest values. This indicates that starch from these genotypes had less ability to withstand heating at high temperatures and the shear stress, which is an important factor in many processes [71]. On the other hand, control durum wheats had significantly lower values for BV, indicating the highest resistance to shearing at high temperature.

The hard hexaploid lines recorded higher pasting temperature values compared to the control lines (*p* < 0.05). These could be due to their higher protein content, which can interfere with starch gelatinization. The increased protein content may form a denser gluten network, raising the resistance to heat and subsequently increasing the temperature required for pasting compared to the control lines.

### 3.6. Solvent Retention Capacity

Significant differences were recorded among the analyzed genotypes in all SRC determinations (*p* < 0.05) (Table 5). The values obtained in the semolina samples were in line with those reported by Labuschagne et al. [6].

SRSCarb is a damaged starch indicator and, indirectly, it is also positively related to grain texture [72]. SRCCarb ranged between 67.99 and 78.92%. Control durum wheats and SD 31 significantly exhibited the highest values of SRCCarb (*p* < 0.05). Higher SRCCarb may be attributed to the presence of higher damaged starch produced during milling [73]. Wheat lines with higher SRCCarb values also show higher d(4,3) values, indicating greater grain hardness.

SRCSuc is associated with gliadin and arabinoxylans content [72]. SRCSuc ranged between 71.74% for SD 39 and 91.05% for control durum wheats. SRCLac is associated with glutenin network formation and gluten strength of flour, because a pH below 7 favors swelling and network formation by gluten polymers relative to polysaccharides [72]. SRCLac values ranged between 65.06 and 76.04%. Control durum wheats and SD 31 had higher SRCLac, which might be due to greater swelling of glutenins that form an extensive gluten network for improved mechanical strength. SRCW ranged from 60.88% for SD 55 to 69.38% for control durum wheats. Statistically significant differences were found among the semolina under study (*p* < 0.05), with the control durum wheat lines presenting higher values than the rest.

### 3.7. Pasta Cooking Properties

Table 6 summarizes the results of cooking properties for pasta samples made from control durum wheats (P-Control durum wheats), hard hexaploid lines (P-SD), and the parent line BIOINTA 3000 GPC (P-B) and Prointa Gaucho (P-PG).

The optimal cooking time (OCT) varied from 7 to 13 min. All hard hexaploid lines of pasta samples showed longer OCT than that found in control durum wheat pasta (*p* < 0.05). A larger D10 indicates larger particles, which take longer to hydrate and gelatinize during cooking, resulting in a longer OCT. The pasta made with the parent lines recorded intermediate OCT values.

CL is used as a main parameter of the pasta cooking performance since it is related to the solid leaching and pasta resistance to disintegration during cooking [74]. In our study, CL values ranged from 5.32 to 7.03% (Table 6), which is within the expected limits (7–8%) for pasta made from durum wheat, indicating good binding of starch and protein components. Similar results were found by Dziki [11], Kaplan Evlice [55], and Suo et al. [75]. Significant differences were found between the CL values of P-control durum wheat semolina, P-B, and P-SD lines (*p* < 0.05). P-34 and P-39 pasta presented the highest CL values, but the values remain within the previously established limits.

SI values ranged from 1.82 to 2.13% (Table 6) in concordance with Bokić et al. [74] and Oduro-Obeng et al. [62]. No significant differences were found for this parameter among the analyzed pasta samples.

WA is an index reflecting the water absorbed by the pasta in cooking conditions. WA values ranged from 162.02 to 202.61% (Table 6) and were in concordance with Bokić et al. [74]. Significant differences were recorded in the WA values among the analyzed pastas. P-control durum pasta and P-B pasta showed the lowest WA values (*p* < 0.05). The hard hexaploid lines recorded higher protein values compared to the control durum wheats. Additionally, the P-SD samples exhibited higher water absorption values. This relationship may be attributed to the fact that higher protein content contributes to a stronger and more elastic gluten network, which helps retain more water within the pasta structure, allowing for greater swelling.

### 3.8. Cooked Pasta Textural Analysis

The texture values of the analyzed pastas are presented in Table 6. The values of hardness and chewiness in the analyzed pasta ranged from 7.66–12.99 N and 4.53–7.57 N, respectively. Similar values were found by Padalino et al. [76] who studied pasta made with durum wheat semolina. Statistically significant differences were recorded among the analyzed pasta for both parameters (*p* < 0.05). Pasta made from SD 39 line recorded the lowest values of hardness and chewiness, while P-control durum wheats and P-B recorded the highest values for both parameters. Pasta with larger particles tends to be less firm, possibly due to a less compact structure and lower cohesion between particles [2]. P-SD pasta exhibited higher hardness and chewiness values compared to commercial pasta made from bread wheat [77], suggesting that the modifications introduced in the experimental lines contributed positively to pasta quality by enhancing its textural properties.

### 3.9. Color of Cooked Pasta

The color of food is an important attribute of food quality. The color of common pasta strongly depends on the properties of raw material (flour or semolina), such as carotenoids and the composition of protein [74].

The L* parameter ranged from 58.02 to 70.78; the a* parameter ranged from 4.50 to 7.91, while the b* parameter had a minimum of 16.64 and a maximum of 26.74 (Table 6). Similar b* values were reported by Cabas-Lühmann and Manthey [78]. P-control durum wheats recorded significantly the highest L* and b* values and the lowest a* values (*p* < 0.05). P-PG did not differ in any parameter from P-SD pasta, especially in the b* parameter, confirming the results previously found in the semolina. P-SD pasta exhibited higher L* and b* values compared to pasta made from common wheat, suggesting an effective transfer of the yellow pigment to the hard hexaploid wheat lines [77]. Generally, cooked pasta samples tend to exhibit lower L* values compared to the raw semolina used as raw material. This reduction in lightness may be attributed to non-enzymatic browning reactions, primarily the Maillard reaction between reducing sugars and amino acids that occur during drying and cooking. These reactions result in the formation of brown pigments, which decrease the L* value. Additionally, the presence of phenolic compounds and oxidative reactions during processing may further contribute to the darkening of the product [48].

### 3.10. Principal Component Analysis

**Figure 3:**
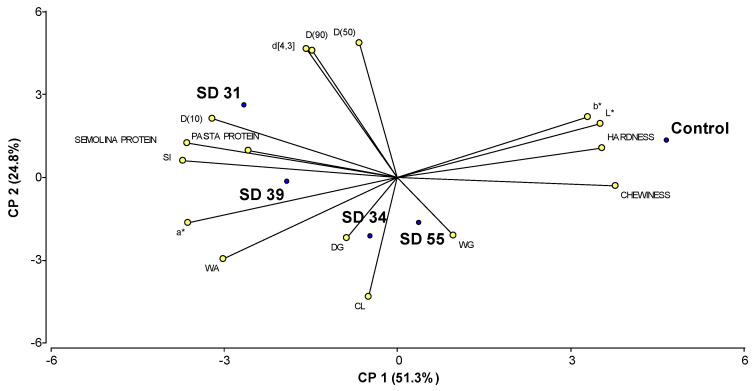
Biplot of Principal Component Analysis of the analyzed wheats (control durum wheats and modified hexaploid wheats). The points represent the cultivars, and the rays correspond to the evaluated characteristics (L*, a*, b* (color values); D(10), D(50), D(90) and d[4,3] (particle size distribution parameters); WG: wet gluten; DG: dry gluten, SI: swelling index; WA: water absorption; CL: cooking loss).

In order to explore the variability among different wheat varieties (control durum wheats and hard hexaploid lines) in terms of certain semolina characteristics and the cooking and texture properties of pasta, a Principal Component Analysis (PCA) was conducted. Figure 3 shows the biplot obtained from the PCA. Each point on the graph corresponds to the genotype under study (control durum wheat and hard hexaploid lines), while the rays represent each of the analyzed characteristics. The direction and length of the rays indicate the loadings of the variables, that is, how each variable contributes to the principal components.

The two axes together explained 76.1% of the variability found among the different cultivars. These two components provide a meaningful representation of the relationships between the variables and the wheat lines, as highlighted in the biplot, where variables with higher eigenvector values exert greater influence on the respective components (Table 7). Principal Component 1 (PC1) explained 51.3% of the variability, and it is primarily associated with texture and color characteristics, showing strong positive contributions from chewability, hardness, and lightness (L*), and negative contributions from SI and color parameter (a*). In contrast, Principal Component 2 (PC2) explained 24.8% of the total variability and it is more related to particle size (D(50), D(90), d[4,3]) and cooking properties, with positive influences from particle size values and negative influences from CL and WA values.

The angles between the variables in the biplot reflect their relationships, with smaller angles indicating stronger positive correlations. Hardness and chewiness have vectors pointing in similar directions, indicating a strong positive correlation. This suggests that firmer pasta tends to be chewier. D(50), D(90), and d[4,3] also show strong positive correlations, as their vectors form acute angles. This implies that larger particle sizes are closely related. On the other hand, CL and WA are negatively correlated with hardness and chewiness, as their vectors point in opposite directions. Similarly, a comparable trend was observed for hardness, chewiness, and protein content. Indeed, studies have reported that protein content in semolina does not always correlate positively with pasta firmness or chewiness. For instance, Padalino et al. [76] demonstrated that higher protein levels (particularly those derived from non-gluten or heat-modified sources) may negatively affect pasta texture. These findings are consistent with the distribution observed in our biplot, where firmness and protein content were located at opposite ends of the principal axis.

The control durum wheats appear to be positioned closer to variables such as L* and b*, and textural properties. Among the hard hexaploid lines, the SD 31 line stands out as distinct, showing strong correlations with variables related to semolina particle size (d[4,3], D(50), D(90), etc.), and protein content. SD 34 and SD 39 lines are positioned closer to variables such as DG and WA. SD 55 line is closer to variables such as WG and CL, and it is also located closer to the control durum wheat, suggesting it shares more similarities with the control.

## 4. Conclusions

This study demonstrated that most hard hexaploid lines recorded values similar to the control durum wheats for total starch, ash, TAx, WEATx, WG, DG content, and GI values. However, the hard hexaploid lines exhibited higher protein content, a critical parameter for ensuring pasta quality. Additionally, color analyses on both semolina and pasta samples confirmed the successful transfer of desirable yellow pigmentation traits, enhancing the visual appeal of the pasta. However, they did not achieve brightness and yellowness values similar to those of the commercial control durum wheat pasta, an aspect that should be improved in future studies. Textural analysis revealed that while some lines exhibited slightly lower hardness and chewiness than durum wheat pasta, the SD 34 line recorded the highest values among the hard hexaploid lines. Moreover, the SD 55 line showed characteristics closely comparable to traditional pasta, including high WG and superior cooking resilience, suggesting its potential as a viable alternative for high-quality pasta production. These findings underscore the potential of marker-assisted selection to enhance bread wheat cultivars for diverse applications, enabling the development of cost-effective and high-quality pasta options in regions with limited access to durum wheat. However, further work is needed either by improving these experimental lines or by developing new ones that allow obtaining wheat lines capable of producing pasta with superior quality, particularly in terms of color and textural properties.

## Figures and Tables

**Table 1:** Cultivar Assessment: Parental lines, control durum wheat varieties, and hard hexaploid wheat lines.

Parent Hexaploid Wheats Lines	Control Durum Wheats Varieties	Hard Hexaploid Wheats Lines
BIOINTA 3000 GPC Linea Lr19 PIN Prointa Gaucho	ACA 1801 F Bonaerense INTA Cariló Bonaerense INTA Facón Bonaerense INTA Quillén	SD 31 SD 34 SD 39 SD 55

**Table 2:** Vitreousness values and particle size distribution of semolina samples.

	D(10) (µm)	D(50) (µm)	D(90) (µm)	d[4.3] (µm)	Span	Vitreousness (%)
Control durum wheats	26.68 ^a^	303.88 ^a^	752.22 ^a^	342.74 ^a^	2.33 ^b^	85.13 ^b^
SD 31	38.00 ^b^	310.46 ^a^	1646.26 ^b^	547.70 ^b^	5.07 ^a^	0 ^a^
SD 34	30.82 ^a^	290.96 ^a^	562.39 ^a^	302.13 ^a^	1.83 ^b^	0 ^a^
SD 39	31.01 ^a^	305.49 ^a^	593.63 ^a^	318.13 ^a^	1.84 ^b^	0 ^a^
SD 55	31.35 ^a^	283.60 ^a^	557.11 ^a^	295.14 ^a^	1.86 ^b^	0 ^a^
BIOINTA 3000 GPC	**	**	**	**	**	1 ^a^
Prointa Gaucho	**	**	**	**	**	4.25 ^a^
Linea Lr19 PIN	**	**	**	**	**	18.88 ^a^

Different letters within the same column represent significant differences between cultivars (*p* < 0.05). D(10) (value of particle size below which 10% of the particles are found), D(50) (value of the particle diameter at 50% in the cumulative distribution), D(90) (value of particle size below which 90% of the particles are found), span (provides the separation range between 10 and 90% of the points, normalized to the midpoint) and d[4,3] (represents the volume-weighted mean diameter of particles). ** cultivar not evaluated for selected characteristic.

**Table 3:** Chemical characteristics of different wheat varieties.

	Ash (%)	TAx (%)	WEAx (%)	Total Starch (%)	Amylose (%)	Protein Content (%)	WG (%)	DG (%)	GI
Control durum wheats	1.21 ± 0.03 ^a^	7.22 ± 0.3 ^a^	0.40 ± 0.05 ^a^	72.95 ± 0.7 ^a^	26.64 ± 0.52 ^b^	9.80 ± 0.12 ^a^	29.63 ± 1.5 ^a^	10.64 ± 0.4 ^a^	88.02 ± 1.2 ^a^
SD 31	1.14 ± 0.05 ^a^	6.58 ± 0.6 ^a^	0.50 ± 0.05 ^a^	69.90 ± 1.5 ^a^	26.96 ± 0.90 ^b^	11.80 ± 0.24 ^c^	29.70 ± 2.1 ^a^	11.49 ± 0.8 ^a^	94.47 ± 2.3 ^a^
SD 34	1.12 ± 0.09 ^a^	6.63 ± 0.8 ^a^	0.46 ± 0.06 ^a^	71.08 ± 1.8 ^a^	20.84 ± 0.95 ^a^	10.94 ± 0.34 ^b^	30.73 ± 2.5 ^a^	11.58 ± 1.0 ^a^	90.47 ± 2.8 ^a^
SD 39	1.03 ± 0.07 ^a^	8.24 ± 0.6 ^a^	0.50 ± 0.05 ^a^	71.66 ± 1.4 ^a^	23.05 ± 0.96 ^a^	10.96 ± 0.24 ^b^	27.06 ± 2.5 ^a^	10.45 ± 0.9 ^a^	93.29 ± 2.5 ^a^
SD 55	1.10 ± 0.07 ^a^	6.95 ± 0.6 ^a^	0.40 ± 0.03 ^a^	70.13 ± 1.4 ^a^	27.55 ± 1.00 ^b^	10.60 ± 0.24 ^b^	31.76 ± 2.5 ^a^	12.09 ± 0.9 ^a^	93.93 ± 2.5 ^a^
BIOINTA 3000 GPC	**	**	**	**	**	10.83 ± 0.40 ^b^	25.96 ± 2.0 ^a^	9.53 ± 0.60 ^a^	94.10 ± 2.6 ^a^

TAx: Total arabinoxylans; WEAx: water extractable arabinoxylans; WG: wet gluten; DG: dry gluten; GI: gluten index. Different letters within the same column represent significant differences between cultivars (*p* < 0.05). ** cultivar not evaluated for selected characteristic.

**Table 4:** Pasting properties on semolina samples.

	PV	Trough	BV	FV	Setback	PT
Control durum wheats	2261.13 ± 63 ^a^	1699.94 ± 43.5 ^b^	561.19 ± 22.4 ^a^	3552.88 ± 83.4 ^b^	1852.94 ± 40.6 ^b^	5.56 ± 0.02 ^a^
SD 31	2866.00 ± 120 ^b^	1704.25 ± 87.1 ^b^	1161.75 ± 44.9 ^c^	3691.75 ± 160 ^b^	1987.50 ± 81.8 ^b^	5.68 ± 0.05 ^b^
SD 34	2382.50 ± 150 ^a^	1464.50 ± 120 ^a^	918.00 ± 63 ^b^	3187.00 ± 150 ^a^	1722.50 ± 90.1 ^a^	5.67 ± 0.05 ^b^
SD 39	2188.50 ± 123 ^a^	1316.75 ± 87.1 ^a^	871.75 ± 44.1 ^b^	2917.75 ± 166 ^a^	1601.00 ± 81.3 ^a^	5.64 ± 0.05 ^b^
SD 55	2558.75 ± 125 ^a^	1736.75 ± 87.1 ^b^	822.00 ± 44.2 ^b^	3635.00 ± 166 ^b^	1898.25 ± 81.4 ^b^	5.77 ± 0.05 ^b^

PV: peak viscosity; BV: breakdown viscosity; FV: final viscosity; PT: pasting temperature. Different letters within the same column represent significant differences between cultivars (*p* < 0.05).

**Table 5:** Solvent Retention Capacity (SRC) values of the analyzed semolina samples.

	SRSCarb	SRCSuc	SRCLac	SRCW
Control durum wheats	78.92 ± 0.60 ^b^	91.05 ± 1.33 ^b^	76.04 ± 1.12 ^b^	69.38 ± 0.73 ^b^
SD 31	76.34 ± 1.19 ^b^	78.54 ± 2.67 ^a^	73.17 ± 2.23 ^b^	65.36 ± 1.40 ^a^
SD 34	72.49 ± 1.68 ^a^	76.48 ± 3.77 ^a^	66.87 ± 3.10 ^a^	64.09 ± 2.10 ^a^
SD 39	72.43 ± 1.19 ^a^	71.74 ± 2.67 ^a^	67.93 ± 2.23 ^a^	63.23 ± 1.40 ^a^
SD 55	70.47 ± 1.19 ^a^	76.48 ± 3.77 ^a^	67.75 ± 2.23 ^a^	63.29 ± 1.40 ^a^

SRCW: water, SRCSuc: sucrose, SRSCarb: sodium carbonate, SRCLac: lactic acid. Different letters within the same column represent significant differences between cultivars (*p* < 0.05).

**Table 6:** Pasta cooking, texture, and color parameters of pasta samples.

	OCT (min)	CL (%)	SI (%)	WA (%)	Hardness (N)	Chewiness (N)	L*	a*	b*
P-Control durum wheats	7 ^a^	5.91 ± 0.15 ^a^	1.82 ± 0.05 ^a^	162.02 ± 3.10 ^a^	12.99 ± 0.28 ^c^	7.57 ± 0.21 ^c^	70.78 ± 0.40 ^c^	4.5 ± 0.16 ^a^	26.74 ± 0.30 ^b^
P-SD 31	12 ^c^	5.65 ± 0.29 ^a^	2.13 ± 0.11 ^a^	182.55 ± 4.30 ^b^	9.06 ± 0.53 ^b^	4.70 ± 0.39 ^a^	59.95 ± 0.80 ^a^	7.47 ± 0.32 ^c^	17.36 ± 0.60 ^a^
P-SD 34	13 ^d^	7.03 ± 0.23 ^b^	1.98 ± 0.15 ^a^	197.14 ± 5.20 ^b^	10.01 ± 0.72 ^b^	5.91 ± 0.53 ^b^	58.02 ± 0.90 ^a^	7.81 ± 0.45 ^c^	17.52 ± 0.80 ^a^
P-SD 39	13 ^d^	6.43 ± 0.29 ^b^	2.06 ± 0.11 ^a^	202.61 ± 5.03 ^b^	7.66 ± 0.48 ^a^	4.53 ± 0.36 ^a^	60.25 ± 0.80 ^a^	7.91 ± 0.32 ^c^	18.89 ± 0.60 ^a^
P-SD 55	13 ^d^	6.02 ± 0.29 ^a^	2.03 ± 0.11 ^a^	185.63 ± 4.50 ^b^	9.33 ± 0.51 ^b^	5.80 ± 0.37 ^b^	62.73 ± 0.80 ^b^	6.61 ± 0.32 ^b^	16.64 ± 0.60 ^a^
P-B	9 ^b^	5.32 ± 0.15 ^a^	1.80 ± 0.05 ^a^	162.38 ± 3.10 ^a^	12.64 ± 0.23 ^c^	6.84 ± 0.41 ^c^	**	**	**
P-PG	**	**	**	**	**	**	59.08 ± 0.60 ^a^	7.42 ± 0.32 ^c^	17.61 ± 0.60 ^a^

OCT: optimal cooking time; WA: water absorption; SI: swelling index; CL: Cooking Loss. P-: pasta made with the specific line. P-B: pasta made from parent line BIOINTA 3000 GPC; P-PG: pasta made from Prointa Gaucho. Different letters within the same column represent significant pasta differences (*p* < 0.05). ** cultivar not evaluated for selected characteristic.

**Table 7:** Eigenvectors corresponding to Principal ComponentAnalysis.

Variables	e1	e2
D(10)	−0.29	0.19
D(50)	−0.06	0.44
D(90)	−0.13	0.42
d[4,3]	−0.14	0.42
WG	0.09	−0.19
DG	−0.08	−0.20
SI	−0.33	0.06
CL	−0.04	−0.39
WA	−0.27	−0.27
Chewiness	0.34	−0.03
Hardness	0.32	0.09
L*	0.32	0.17
a*	−0.33	−0.15
b*	0.30	0.20
Semolina protein	−0.33	0.11
Pasta protein	−0.23	0.09

L*, a*, b* (semolina color values); D(10), D(50), D(90) and d[4,3] (particle size distribution parameters); WG: wet gluten; DG: dry gluten, SI: swelling index; WA: water absorption; CL: cooking loss). e1 and e2 represent the first and second principal components (PC1 and PC2), respectively.

## Data Availability

The original contributions presented in the study are included in the article, further inquiries can be directed to the corresponding author.

## Abbreviations

The following abbreviations are used in this manuscript:

MAS	Marker-assisted selection
TAx	Total arabinoxylans
WEAx	Water-extractable arabinoxylans
WG	Wet gluten
DG	Dry gluten
GI	Gluten index
PT	Pasting temperature
PV	Peak viscosity
FV	Final viscosity
BV	Breakdown viscosity
SRCW	Water solvent retention capacity
SRCSuc	Sucrose solvent retention capacity
SRSCarb	Sodium carbonate solvent retention capacity
SRCLac	Lactic acid solvent retention capacity
OTC	Optimal cooking time
WA	Water absorption
SI	Swelling index
CL	Cooking loss
PCA	Principal Component Analysis
